# Comparison of blood metal ion levels in scoliosis patients following instrumented spinal fusion with cobalt-chromium and titanium alloy rods

**DOI:** 10.1038/s41598-025-99065-x

**Published:** 2025-05-13

**Authors:** Shota Tamagawa, Hidetoshi Nojiri, Tatsuya Sato, Takehisa Matsukawa, Yasuhiro Homma, Hiromitsu Takano, Muneaki Ishijima

**Affiliations:** 1 Department of Orthopaedics, Juntendo University Faculty of Medicine, 2-1-1 Hongo, Bunkyo-Ku, Tokyo, 113-8421 Japan; 2 Department of Medicine for Orthopaedics and Motor Organ, Juntendo University Graduate School of Medicine, Tokyo, Japan; 3 Department of Epidemiology and Environmental Health, Juntendo University Faculty of Medicine, Tokyo, Japan; 4 Department of Forensic Medicine, Juntendo University Faculty of Medicine, Tokyo, Japan; 5 Laboratory of Hygienic Chemistry, Juntendo University Faculty of Pharmacy, Chiba, Japan

**Keywords:** Blood metal ion, Cobalt, Chromium, Titanium, Scoliosis, Instrumented spinal fusion, Surgery, Musculoskeletal abnormalities, Biomarkers

## Abstract

Elevated blood metal ion levels have been reported in patients following instrumented spinal fusion. However, the relationship between blood metal ion levels and clinical and radiographic outcomes remains elusive. The purpose of this study was to compare blood metal ion levels in patients with scoliosis undergoing corrective fusion surgery utilizing either cobalt-chromium (CoCr) or titanium (Ti) alloy rods, as well as preoperative scoliosis patients. Additionally, we investigated patient- and surgery-related factors associated with blood metal ion levels. This retrospective cohort study included 59 patients with CoCr rods (CoCr group), 29 patients with Ti alloy rods (Ti group), and 17 preoperative patients (control group). Blood samples were collected at a mean of 63 months postoperatively for the CoCr group and 113 months postoperatively for the Ti group. Blood levels of Co, Cr, and Ti were measured by inductively coupled plasma mass spectrometry. Blood Co levels were significantly elevated in the CoCr group compared to the control group (p = 0.021), with no significant difference observed between the CoCr and Ti groups (CoCr group: 0.28 μg/L, Ti group: 0.21 μg/L, control group: 0.20 μg/L). However, blood Cr and Ti levels were found to be below the limit of quantification in all 105 individuals. Furthermore, a significant negative correlation was observed between blood Co levels and the postoperative follow-up duration. These findings underscore the medium-term safety rather than the concerns regarding blood metal ion levels following corrective fusion for scoliosis; however, prolonged and meticulous follow-up is imperative.

## Introduction

Elevated blood metal ion levels and the deposition of metal debris in peri-implant tissues have been documented following metal-on-metal joint replacement and instrumented spine fusion^1–5^. All surgical implants are subject to some degree of wear and corrosion in the body, resulting in the liberation of metal particles from the implants into the adjacent tissues and eventually into the bloodstream^6,7^. Additionally, macrophage activation induced by metal debris can exacerbate inflammation and bone resorption, potentially precipitating implant failure^8–10^. Metallosis, a common occurrence after orthopedic implant procedures, is typically asymptomatic and clinically insignificant; however, it may occasionally necessitate revision arthroplasty^11,12^. Nevertheless, the phenomenon of metallosis in instrumented spine surgery remains relatively understudied.

Instrumented spinal fusion is a standard treatment for patients with various spinal pathologies and has witnessed an increasing trend in recent years^13^. Specifically, scoliosis surgery is predominantly performed in the early life stages, necessitating extensive instrumentation with implants typically retained for the duration of the patient’s life^14^. Consequently, there is considerable concern surrounding postoperative alterations in blood metal ion levels and their potential long-term repercussions on the human body.

A recent systematic review highlighted increased blood metal ion levels following instrumented spinal fusion, specifically noting elevated chromium (Cr) levels associated with stainless steel implants and titanium (Ti) levels linked to Ti implants^14^. In addition, findings from the meta-analysis conducted by Burgos et al. indicated a significant increase in blood metal ion levels, particularly Ti and Cr, following spinal fusion surgery compared to both preoperative levels and healthy controls^15^. However, no definitive association between blood metal ion levels and clinical and radiographic outcomes has been established.

The purpose of this study was to evaluate blood metal ion levels in patients following scoliosis surgery utilizing either cobalt-chromium (CoCr) or Ti alloy rods, and in preoperative scoliosis patients. Additionally, the study aimed to investigate patient- and surgery-related factors potentially correlated with blood metal ion levels.

## Materials and methods

### Study design and patient identification

**Fig. 1:**
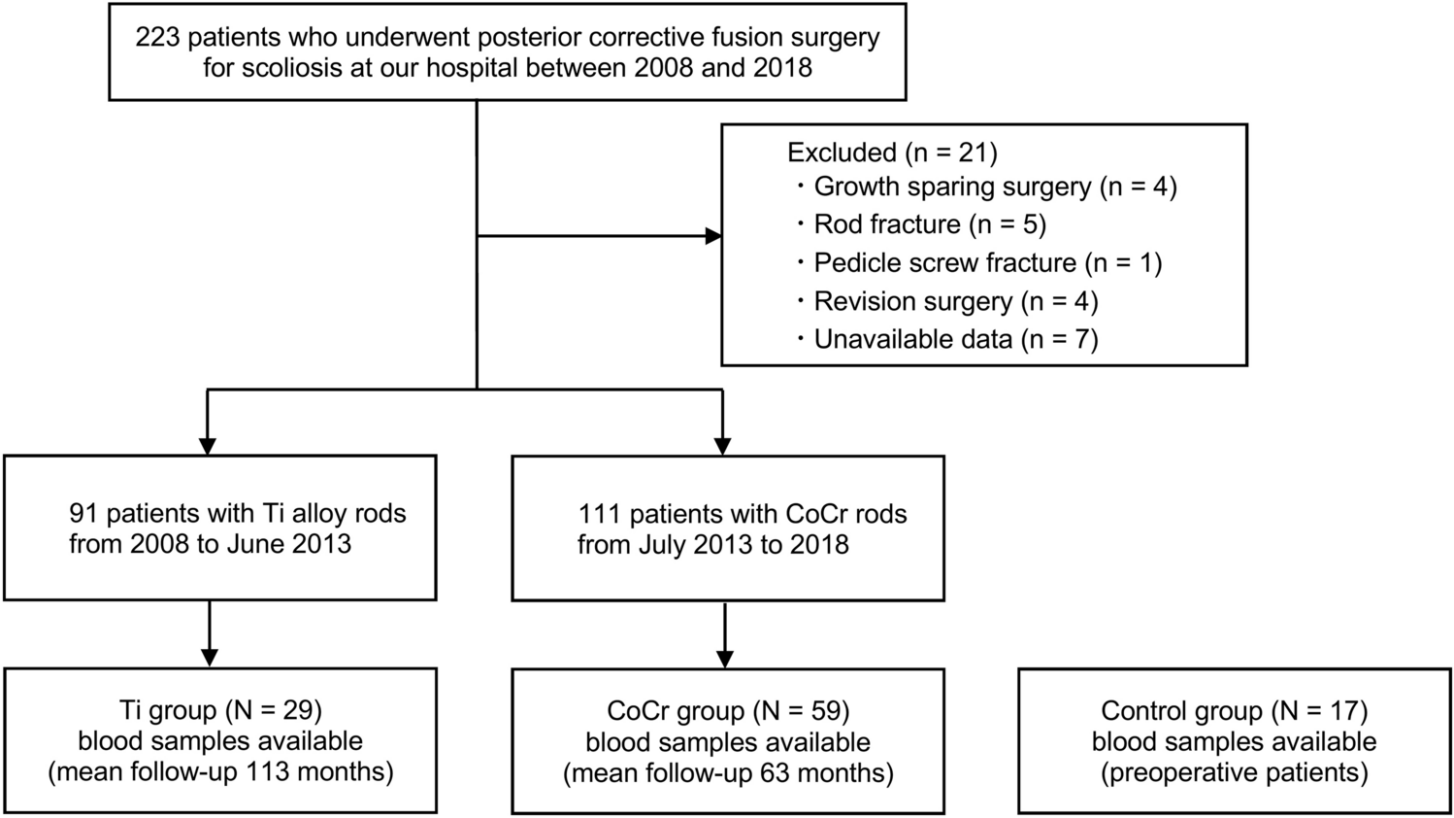
Patient recruitment flowchart.

This retrospective cohort study was approved by the institutional review board of our university hospital (H19-0284). All research was performed in accordance with the Declaration of Helsinki. Informed consent was obtained from all individual participants or legal guardians included in the study. Patients who underwent posterior corrective fusion surgery for scoliosis at our university hospital between 2008 and 2018 were enrolled in this study. Inclusion criteria were scoliosis patients younger than 40 years at the time of surgery, with a minimum of 2 years of postoperative follow-up. Exclusion criteria included growth sparing surgery, implant failure, revision surgery, and unavailable data. Our hospital employed 6.35-mm diameter Ti alloy rods with Ti alloy screws (CD HORIZON Legacy; Medtronic) from 2008 to June 2013, and 6.0-mm diameter CoCr rods with Ti alloy screws (CD HORIZON Solera; Medtronic) from July 2013 to 2018. After obtaining informed consent, we identified 59 patients with CoCr rods (CoCr group), 29 patients with Ti alloy rods (Ti group), and 17 preoperative patients (control group) whose blood metal ion levels could be measured (Fig. 1).

### Radiographic measurements

All patients underwent routine upright posteroanterior and lateral radiographs before and after surgery. The degree of the main curve was assessed using the Cobb angle protocol. The correction rate was calculated as the difference between the preoperative Cobb angle and the postoperative follow-up Cobb angle, divided by the preoperative Cobb angle on the upright radiograph, and then multiplied by 100 (%). The number of fixed vertebrae, pedicle screws, hooks, and crosslinks were counted. The number of rod attachments was defined as the number of connections between the rod and the screws or hooks. The total rod length (mm) was calculated by summing the lengths of the left and right rods, while the total rod surface area (mm^2^) was calculated by summing the surface areas of the left and right rods (twice the rod base area plus the rod side area). Radiographic measurements were performed by the third author, who was not involved in the statistical analysis.

### Blood metal ion measurement

Blood concentrations of Co, Cr, and Ti ions were determined by inductively coupled plasma mass spectrometry (ICP-MS) using an Agilent 8800 instrument (Agilent Technologies, Tokyo, Japan). Sample preparation and analysis were performed according to a previously described method^16^. Prior to ICP-MS analysis, blood samples were digested using a microwave digestion system (ETHOS PLUS, Milestone, Sorisole, Italy) with 68% nitric acid and 35% hydrogen peroxide (Ultrapure Grade, Tama Chemicals, Tokyo, Japan). The digested samples were then diluted with 0.5% nitric acid containing 20 μg/mL yttrium as an internal standard. Quantification was carried out using an internal standard method, utilizing the mass-to-charge ratios (m/z) of 89 for yttrium and 59, 52, and 50 for Co, Cr, and Ti, respectively. To ensure the accuracy of metal ion measurements in blood, quality control samples from Seronorm Trace Elements Whole Blood Control Level-2 and Level-3 (Sero, Billingstad, Norway) were analyzed. The quantitative results obtained by the internal standard method were compared with those from the standard addition method to validate the consistency between the two approaches, thereby confirming the accuracy of the measurements. The limit of quantification (LOQ) was defined as 10 times the standard deviation of the blank sample’s signal intensity, and it was expressed in concentration units (μg/L). In this study, the LOQ for Co ranged from 0.07 to 0.13 μg/L depending on the day of analysis, while the maximum LOQ values for Cr and Ti were 6.9 and 17.9 μg/L, respectively. Data below the detection limit were set to the LOQ.

### Statistical analysis

All statistical analyses were performed using GraphPad Prism version 9.5.1 (GraphPad Software, CA, USA). Data are presented as mean ± SD. Normality of continuous data distribution was assessed by the Shapiro–Wilk test. For patient baseline characteristics, the chi-squared test was employed for categorical variables, while differences in continuous variables between two groups were assessed using the two-tailed unpaired Student’s t-test or Mann–Whitney U test, as appropriate. Differences among three groups were analyzed by one-way ANOVA, followed by Tukey’s multiple comparisons test or Kruskal–Wallis, followed by Dunn’s multiple comparisons test. Spearman’s rank correlation coefficient was utilized for correlation analysis between blood Co levels and patient demographics. P < 0.05 was considered a statistically significant difference.

## Results

### Patient demographics and radiographic data

Descriptive data for each group are shown in Table 1. The mean age at surgery and the postoperative follow-up duration were significantly different between the CoCr and Ti groups (p = 0.003 and p < 0.001, respectively). All groups exhibited a predominance of adolescent idiopathic scoliosis (AIS) as the primary etiology. Among other etiologies, the CoCr group included 2 patients with congenital scoliosis, 3 patients with neuromuscular scoliosis, and 2 patients with syndromic scoliosis; the Ti group comprised 2 patients with neuromuscular scoliosis and 2 patients with syndromic scoliosis; and the control group encompassed 1 patient with neuromuscular scoliosis and 1 patient with syndromic scoliosis.

**Table 1:** Patient demographics (Data are expressed as mean ± SD).

		CoCr rod (n = 59)	Ti rod (n = 29)	Control (n = 17)
Patient characteristics				
Age (years)		18.3 ± 5.1*	16.2 ± 3.9*	16.9 ± 3.2
Sex, n (%)	Male	11 (18.6)	3 (10.3)	2 (11.8)
	Female	48 (81.4)	26 (89.7)	15 (88.2)
BMI (kg/m^2^)		19.6 ± 2.4	19.5 ± 3.1	19.5 ± 2.1
Etiology, n (%)	Idiopathic	52 (88.1)	26 (89.7)	15 (88.2)
	Others	7 (11.9)	3 (10.3)	2 (11.8)
Postoperative follow-up duration (months)		63 ± 17*	113 ± 16*	N/A
Radiographic measures				
Preoperative main curve (degree)		61 ± 14	61 ± 10	52 ± 12*
Main curve at follow-up (degree)		24 ± 10	25 ± 9	N/A
Correction rate at follow-up (%)		60.9 ± 10.1	60.1 ± 12.1	N/A
Number of fixed vertebrae		12.4 ± 1.7	13.2 ± 1.6	N/A
Number of pedicle screws		17.1 ± 3.2*	11.5 ± 2.9*	N/A
Number of hooks		1.5 ± 1.4*	5.3 ± 1.7*	N/A
Number of rod attachments		18.7 ± 2.7*	16.9 ± 2.4*	N/A
Number of crosslinks		2.0 ± 0.7	1.9 ± 0.4	N/A
Total rod length (mm)		691 ± 101*	747 ± 72*	N/A
Total rod surface area (mm^2^)		131 ± 19*	149 ± 14*	N/A
Surgical characteristics				
Operative time (min)		405 ± 106	394 ± 61	N/A
Estimated blood loss (ml)		1303 ± 918*	1849 ± 1103*	N/A

* Significant difference from other groups.

Regarding radiographic parameters, the mean Cobb angles of the preoperative main curve were significantly smaller in the control group compared to the surgical group (p = 0.001). The mean Cobb angles of the main curve and the correction rate at follow-up, as well as the number of fixed vertebrae were similar in both groups. The number of pedicle screws was significantly higher in the CoCr group than in the Ti group (p < 0.001), while the number of hooks was significantly lower in the CoCr group than in the Ti group (p < 0.001), resulting in 18.7 ± 2.7 rod connections in the CoCr group and 16.9 ± 2.4 in the Ti group (p = 0.006). In addition, the total rod length and total rod surface area were significantly greater in the Ti group compared to the CoCr group (p = 0.009 and p < 0.001, respectively).

The operative time was comparable in both groups, whereas estimated blood loss was significantly higher in the Ti group compared to the CoCr group.

### Blood metal ion levels

**Fig. 2:**
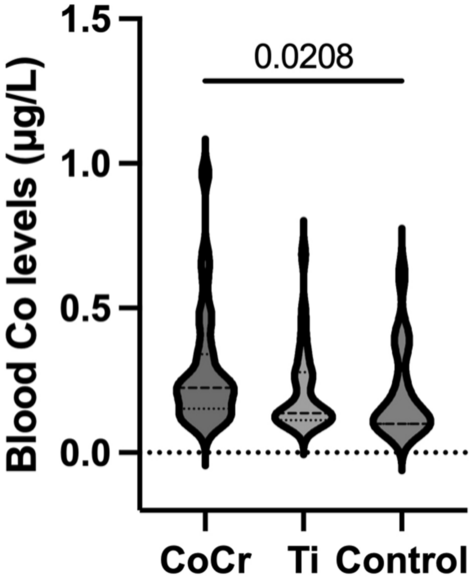
Violin plot of blood Co levels in each group.

**Fig. 3:**
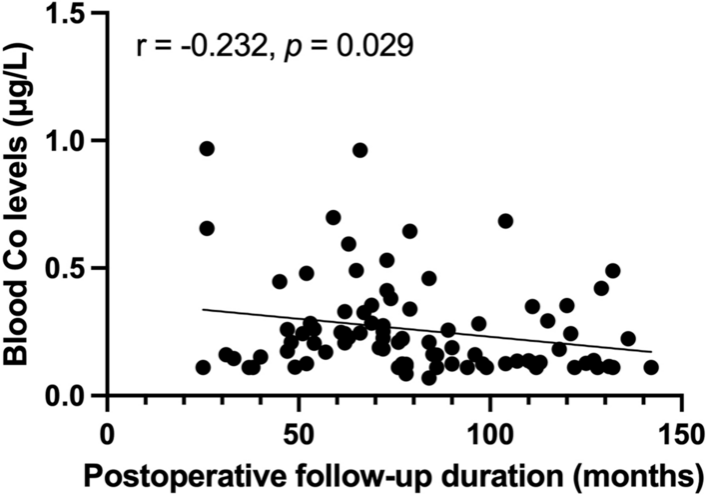
Spearman’s rank correlation coefficient between blood Co levels and postoperative follow-up duration.

Fifty-two of the 59 patients (88.1%) in the CoCr group, 21 of the 29 patients (72.4%) in the Ti group, and eight of the 17 patients (47.1%) in the control group showed blood Co levels above the LOQ (0.07–0.13 μg/L). Blood Co levels measured 0.28 ± 0.20 μg/L in the CoCr group, 0.21 ± 0.14 μg/L in the Ti group, and 0.20 ± 0.15 μg/L in the control group, respectively. Blood Co levels were significantly higher in the CoCr group compared to the control group (p = 0.021); however, there was no significant difference between the CoCr and Ti groups (Fig. 2). In addition, none of the 105 individuals in our study exhibited Co levels above 1.0 μg/L, indicating excessive Co exposure. In this study, blood Cr and Ti levels were found to be below the detection limit in all 105 individuals. Notably, a significant negative correlation was observed between blood Co levels and postoperative follow-up duration (Fig. 3). No other patient characteristics or surgery-related factors exhibited significant correlations with blood Co levels (Table 2).

**Table 2:** Spearman’s rank correlation coefficient between blood Co levels and patient demographics.

	r	*p* value
Age (years)	− 0.001	0.995
BMI (kg/m^2^)	0.060	0.578
Postoperative follow − up duration (months)	− 0.232	0.029*
Preoperative main curve (degree)	0.184	0.085
Main curve at follow − up (degree)	0.065	0.550
Correction rate at follow − up (%)	− 0.001	0.992
Number of fixed vertebrae	0.005	0.963
Number of pedicle screws	0.083	0.441
Number of hooks	− 0.106	0.327
Number of rod attachments	0.033	0.763
Number of crosslinks	0.067	0.534
Total rod length (mm)	− 0.042	0.699
Total rod surface area (mm^2^)	− 0.087	0.423
Operative time (min)	0.113	0.295
Estimated blood loss (ml)	0.010	0.929

## Discussion

In this study, blood Co levels were significantly elevated in the CoCr rod group compared to the non-surgical group, although no significant difference in blood Co levels was observed between the CoCr and Ti alloy rod groups in patients more than 2 years after posterior corrective fusion surgery for scoliosis. In addition, a notable negative correlation was found between blood Co levels and the postoperative follow-up duration, suggesting a potential decrease in blood Co levels over time following fusion surgery.

A limited number of studies have investigated blood Co levels in patients with spinal deformity following instrumented spinal fusion. Sørensen et al. reported that serum Co and Cr levels were comparable in patients with AIS 25 years after Harrington rod instrumentation or bracing^17^. Additionally, a case–control study by Fell et al. demonstrated that serum Co levels were not significantly different in scoliosis patients who underwent instrumented fusion with either stainless steel or Ti implants compared to sex- and age-matched non-surgically treated individuals^5^. Furthermore, they observed a negative correlation between the duration of implantation and serum Cr levels in 71 scoliosis patients with steel implants, but no such correlation was found with Co levels. On the other hand, a prospective study of patients undergoing spinal deformity surgery with repeated testing for circulating metal ions by Cundy et al. showed that Co levels exhibited a gradual increase, reaching a peak at 30 days post-implantation, followed by a gradual decline. However, even after two years, Co levels remained 1.74 times higher than the preoperative baseline level, albeit with a decreasing trend over time^18^. This study revealed a significant increase in blood Co levels in patients treated with CoCr rods compared to the non-surgical group. However, it is noteworthy that the observed difference between the two groups was minimal (CoCr group: 0.28 μg/L vs. control group: 0.20 μg/L) and lacked clinical significance.

Co toxicity resulting from elevated blood Co levels has been associated with various systemic complications, including hematological, neurological, cardiovascular, and endocrine deficits^19,20^. However, there are currently no universally accepted criteria defining a “Co level threshold” above which toxic effects are definitively known to occur, necessitating therapeutic intervention. Although adverse health effects are unlikely to manifest at blood Co levels below 100 μg/L in healthy individuals, symptomatic toxic reactions have been documented at much lower levels in cases of malfunctioning metal-on-metal hip implants^21,22^. This phenomenon may be attributed to underlying diseases that can heighten an individual’s susceptibility to Co toxicity. Moreover, the American Conference of Governmental Industrial Hygienists (ACGIH) suggests that blood Co levels exceeding 1.0 μg/L may indicate potential environmental or occupational overexposure^23^. None of the 105 individuals in our study exhibited Co levels above 1.0 μg/L. In addition, no adverse events indicative of Co toxicity were observed in the cohort examined. Furthermore, given the trend of decreasing blood Co levels during postoperative follow-up, the findings of this study underscore the emphasis on medium-term safety rather than concerns regarding blood metal ion levels following corrective fusion for scoliosis. Nevertheless, Tamagawa et al. reported two cases of scoliosis where blood Co levels increased following rod fracture, subsequently decreasing over time after rod replacement^24^. Since patients with implant failures such as rod fracture or screw loosening were excluded from this study, the long-term safety implications of leaving these patients untreated remain uncertain and warrant further investigation in subsequent studies.

Several studies have reported elevated blood Cr and Ti levels after instrumented spinal fusion surgery^3,18,25,26^. Cundy et al. demonstrated that abnormally elevated serum Cr levels were detected in 37% of patients following posterior spinal arthrodesis with stainless steel instrumentation^3^. A prospective study by Cundy et al. also indicated that serum Cr levels were elevated during and immediately after scoliosis surgery, but returned to baseline levels by 30 days postoperatively^26^. Additionally, another prospective longitudinal study by Cundy et al. revealed that children with spinal implants for deformity correction exhibited persistently elevated serum Ti levels at two years postoperatively, with levels reaching 5.14 times baseline^18^. However, all of the elevated serum metal ion levels detected were subclinical. In the present study, blood Cr and Ti levels were undetectable, possibly influenced by the longer duration since surgery compared to previous studies, with blood sampling obtained at 63 ± 17 months in the CoCr group and 113 ± 16 months in the Ti group. Taken together, the medium-term safety in patients undergoing corrective fusion for scoliosis with any implant material appears to be preserved in terms of blood metal ion levels. Furthermore, the lack of a standardized method for measuring blood metal ion levels complicates comparisons between different studies and remains a challenge for the future.

A previous study identified several significant predictors of serum metal ion levels in children undergoing instrumented spinal fusion with Ti alloy. These predictors included time since surgery, surgical approach (anterior rather than posterior), number of fused vertebrae, number of pedicle screws inserted, the total rod length, and the total implant surface area^25^. In this study, only the postoperative follow-up duration showed a significant negative correlation with blood Co levels, whereas other factors did not exhibit significant correlations. In line with our findings, Rackham et al. demonstrated a decrease in serum Cr levels over time since surgery in patients with AIS who underwent posterior instrumented spinal fusion with stainless steel implants^4^. Additionally, Fell et al. observed a negative correlation between the duration of implantation and serum Cr levels in 71 scoliosis patients undergoing instrumented fusion with steel implants, but no such correlation was found with Co levels^5^.

This study has several limitations. First, as a single institution retrospective study, our results may not be generalizable to the larger population of scoliosis patients undergoing corrective fusion surgery. Second, the sample size of each group is different. Third, the follow-up duration for the CoCr and Ti groups are different due to modifications in surgical procedures at our institution. Fourth, the lack of baseline data for each patient precludes the assessment of longitudinal changes over time. Therefore, a larger scale prospective study is warranted in the future. Finally, this study comprised patients with a mean postoperative duration of 6.7 years and excluded cases of implant failure. Consequently, the results might differ in cases with longer follow-up or in the presence of implant failure.

In conclusion, this study revealed a marginal elevation in blood Co levels in patients with scoliosis undergoing posterior corrective fusion with CoCr rods compared to non-surgically treated individuals with scoliosis. However, no significant difference in blood metal ion levels was observed between the use of CoCr or Ti alloy rods. Furthermore, a notable negative correlation was identified between blood Co levels and the postoperative follow-up duration. These findings emphasize the medium-term safety rather than concerns regarding blood metal ion levels following corrective fusion for scoliosis; however, longer and careful follow-up is essential.

## Data Availability

All data needed to evaluate the conclusions are present in the main text. Additional requests can be made to the corresponding author upon reasonable request.
